# How are global health policies transferred to sub-Saharan Africa countries? A systematic critical review of literature

**DOI:** 10.1186/s12992-022-00821-9

**Published:** 2022-02-23

**Authors:** Walter Denis Odoch, Flavia Senkubuge, Ann Bosibori Masese, Charles Hongoro

**Affiliations:** 1 School of Health Systems and Public Health (SHSPH), Faculty of Health Sciences, University of Pretoria, Gauteng Province, Pretoria, 0028 South Africa; 2 Afya Research and Development, P.O. Box 21743, Plot 2703, Block 208, Bombo Rd, Kampala, Uganda; 3 Developmental, Capable and Ethical State (DCE) Division, Human Sciences Research Council of South Africa Private Bag X41, Pretoria, 0001 South Africa

**Keywords:** Policy transfer, Health, Sub-Saharan Africa, Review of literature

## Abstract

**Background:**

Most sub-Saharan Africa countries adopt global health policies. However, mechanisms with which policy transfers occur have largely been studied amongst developed countries and much less in low- and middle- income countries. The current review sought to contribute to literature in this area by exploring how health policy agendas have been transferred from global to national level in sub-Saharan Africa. This is particularly important in the Sustainable Development Goals (SDGs) era as there are many policy prepositions by global actors to be transferred to national level for example the World Health Organization (WHO) policy principles of health financing reforms that advance Universal Health Coverage (UHC).

**Methods:**

We conducted a critical review of literature following Arksey and O’Malley framework for conducting reviews. We searched EBSCOhost, ProQuest, PubMed, Scopus, Web of Science and Google scholar for articles. We combined the concepts and synonyms of “policy transfer” with those of “sub-Saharan Africa” using Boolean operators in searching databases. Data were analyzed thematically, and results presented narratively.

**Results:**

Nine articles satisfied our eligibility criteria. The predominant policy transfer mechanism in the health sector in sub-Saharan Africa is voluntarism. There are cases of coercion, however, even in the face of coercion, there is usually some level of negotiation. Agency, context and nature of the issue are key influencers in policy transfers. The transfer is likely to be smooth if it is mainly technical and changes are within the confines of a given disease programmatic area. Policies with potential implications on bureaucratic and political status quo are more challenging to transfer.

**Conclusion:**

Policy transfer, irrespective of the mechanism, requires local alignment and appreciation of context by the principal agents, availability of financial resources, a coordination platform and good working relations amongst stakeholders. Potential effects of the policy on the bureaucratic structure and political status are also important during the policy transfer process.

**Supplementary Information:**

The online version contains supplementary material available at 10.1186/s12992-022-00821-9.

## Background

Global policy agenda is comprised of those issues in which international and national actors pay particular attention – and it changes over time [1]. In terms of global agenda development process, in the intergovernmental governance system of the United Nations (United National General Assembly (UNGA)) and its agencies, deliberations on a particular issue may result in a convention, treaty, declaration, agreement, resolution or charter which countries are expected to adopt to guide implementation of specific initiatives. In the development of global agendas, in addition to governments, other actors may also play critical roles. For example, epistemic communities and other non-state actors including civil society organizations (CSOs) may advance and/or advocate for actions to be taken to address an issue of concern [2–4].

Many national health policy responses are guided by ideas marketed and/or promoted by international organizations [4–6]. This is particularly so in sub-Saharan Africa where there in greater reliance on international organizations for standards, technical assistance and financial support [4–6]. As a percentage of total health expenditure, the external funding accounts on average 24% in the WHO African region countries, but can be as high as 74% (Malawi) [7]. For programmatic diseases ((Human Immunodeficiency Virus (HIV), Malaria and (Tuberculosis) TB)) external funding account for over 80% of total funding, for example in eastern and southern Africa when South Africa is excluded, only 20% of the HIV response is funded domestically [8]. Therefore, global agendas are bound to influence national development processes and financial flow, thereby shaping national public policy prioritization [9–12].

Global agenda once adopted at the international level, be it as a resolution, convention, treaty or declaration is usually taken up at the national level through policy transfer [13, 14]. Dolowitz and Marsh refers to such policy transfer as the occurrence of, and processes involved in, the development of programmes, policies, administrative arrangements, institutions and ideas in one political and/or social system based upon the ideas, institutions, programmes and policies emanating from other political and/or social systems [15] (p. 5). In this review, we shall refer to policy transfer as the development of national level health programs or policies based on a global health policy agenda.

Most policy transfer studies analyze transfers among developed countries [16] and in particular, “health is not usually directly analyzed in *most* policy transfer literature” [4] (p.191). There are few policy transfer studies on developing countries, yet they present different issues in policy transfers compared to developed countries [16]. Marsh and Sharman contends that developing countries provide a powerful testing ground for confirming existing policy transfer hypotheses or developing new ones as well as examining the relationship between policy transfer and effectiveness [17]. For developing countries, policy transfer studies are particularly important in the era of the sustainable development goals (SDGs). This is because there are many policy ideas on how to achieve health targets including UHC under goal 3, with anticipated lots of policy learning and adoption.

Therefore, this review sought to firstly contribute to scholarship in the policy transfer field by reviewing how global policies are transferred to sub-Saharan Africa countries with a focus on the health sector. The health sector was chosen because it is an area neglected in policy transfer studies [4], and in most sub-Saharan Africa, the sector heavily relies on normative and other guidelines promoted by international organizations. The reliance on guidelines by international organizations can be seen in a number of countries in sub-Saharan Africa including Uganda, Kenya, Zambia and South Africa that have been reforming their health financing towards achieving UHC target of the SDG declaration [18–23]. The health financing reform principles being adopted are based on the global norm as advanced by the WHO in its guidance of reforms that advance UHC [24, 25]. Secondly, this review sought to highlight lessons that can be considered by actors at national level as they seek to adopt the WHO policy principles of reforms for UHC from other global health policy transfers that have been documented in similar context in sub-Saharan Africa.

### Policy transfer theories, actors and context

In theory, mechanisms with which policy transfer occurs may be voluntary or coercive. The voluntary mechanism entails learning, competition, and mimicry while coercion may be through force or other tools such as conditionality on access to development funding [17, 26, 27]. In learning, a government adopts a foreign institution’s approach and practice rationally with the view that it will produce more efficient and effective policy or program outcomes through lessons drawing. In mimicry, a country copies a foreign model not based on technical or rational thinking, rather on account of symbolic or normative factors such as being perceived as advanced, progressive or because it is a model advanced by an international organization. Coercion involves powerful entities such as a multilateral organization or a high-income country providing support to a lower income country based on fulfillment of some conditions such as adoption of certain policies. While in competition, a country adopts certain policies so that it is not at a disadvantage compared to other countries.

The transfer can be to varying degrees; emulation (adaptation), copying, hybridization and/or synthesis and inspiration [26]. In emulation, a policy from another setting is adapted or modified usually to suit the local context while in copying the policy is usually transferred as it is without modification [26]. In hybridization, policies from a number of settings are used to inform a policy while in inspiration, policy elsewhere triggers or motivates policy development in a learning country [26].

Policy transfer can be multidimensional and multilevel i.e.; global, international and transnational, domestic and inter-organizational [28, 29]. Based on these levels, Dolowitz and Marsh identified 30 permutations of possible policy transfer pathways [27]. This review is concern with the global and/or international to national level policy transfer.

Bennet et al. [30] suggests that understanding policy transfers necessitate understanding of both the actors and their motivation in the process. Global to national policy transfer usually involves actors at global/international and national level, with variable nature and degrees of power which also varies with the stage of the process. The various forms of power usually at display include technical expertise and knowledge, financials, networking capability, legitimacy/moral imperative, access to decision makers, authority, charisma, etc. [31] However, the actions by the various actors during the policy transfer processes are tempered by contextual factors. The contextual factors constrain or privilege the actors’ actions [15]. Contextual factors may be social, political, and economic in nature. Leichter cited by Buse and colleagues [31] categorized contextual factors into situational, structural, cultural and exogenous factors.

## Methodology

### Study design and review question

We conducted a critical review of literature with a focus on exploring the mechanisms with which global health policy agendas are transferred to national level in sub-Saharan Africa and the role of actors and context in the transfer process.

We adapted Arksey and O’Malley framework for reviews [32, 33]. We defined the review questions; identified and selected the studies; abstracted the data; and synthesized and interpreted the results. The review questions were a) What policy transfer mechanism was at play in the policy transfer process for the identified global health policy agenda to a sub-Saharan African country, b) Who were the key actors and what role did they play in the policy transfer process? c) What was the role of contextual factors in the transfer process? d) Was the policy transfer successful? Defining what is considered policy transfer success or failure remains an area of contestation [17, 34], and delving into these argumentations is beyond the scope of the current review. For the current study, we considered policy transfer as successful when a national health policy or program was developed based on the global health policy agenda and failed if no national policy or program guidelines were developed.

### Criteria for considering studies for the review

All study designs were considered for this review. The inclusion criteria were: - the article is an empirical study, article describes a policy transfer of global or international health agenda, the policy transfer study is on a sub-Saharan African country and the article is published in the English language. We excluded policy transfer between or among specific groups of countries, articles purely on theoretical issues around policy transfer, and studies on policy transfer in developed (high-income) and non-Sub-Sharan Africa countries.

### Search methods for identification of studies

We searched google scholar and databases: EBSCOhost, ProQuest, PubMed, Scopus, and Web of Science. The search was conducted between 15th June and 25th July 2021, we did not restrict our search by year of study or publication. In searching google scholar we used the term policy transfer and manually screened up to the tenth page of the search for the term to identify a relevant article based on our inclusion and exclusion criteria. In searching the electronic databases, we combined the concepts and synonyms of policy transfer with those of sub-Saharan Africa using Boolean operators “OR” and “AND”. An example from PubMed is provided in additional file 1. In addition, we screened the reference lists of included studies from the databases for additional eligible studies.

### Data collection, extraction and analysis

#### Selecting studies

**Fig. 1:**
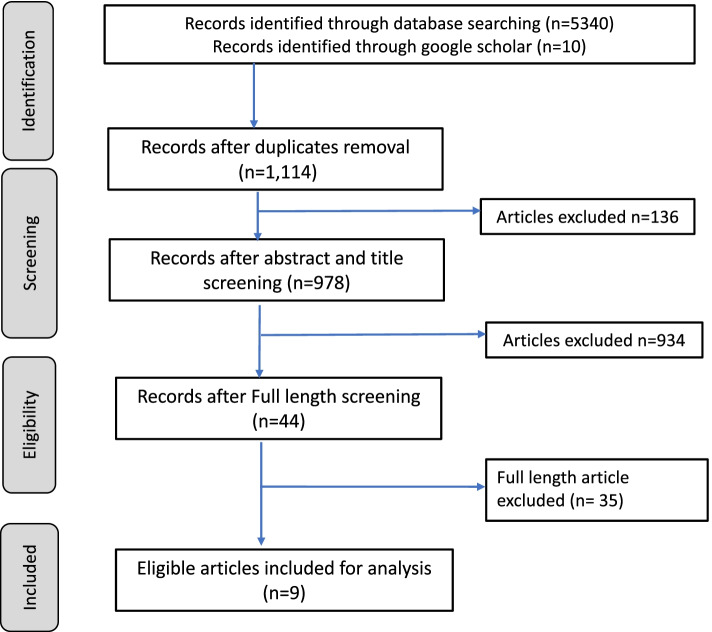
Article selection flow chart

All retrieved articles from the databases were exported to EndNote X9 [35], where duplicates were removed. The titles and abstracts of identified articles were screened for potential eligibility. Full text of articles judged as potentially eligible were retrieved. The articles retrieved were screened in detail for eligibility using a standardized screening form (Additional file 1). Screening and selection of articles were conducted independently by WDO and ABM and disagreements were resolved by consensus. The number of studies included and excluded are as illustrated in the flow diagram (Fig. 1).

#### Data extraction

The study characteristics extracted included bibliographic details of the study (author, year of publication), objectives (purpose of the study), setting (country); global health policy agenda examined, policy transfer mechanisms, policy transfer strategy, the actors and contextual factors.

#### Synthesis and interpretation of results

We used NVIVo and a thematic framework table to facilitate analysis. Thematic framework synthesis approach was used in the analysis. Thematic framework synthesis is a qualitative approach that involves selecting, recording and categorizing key issues and themes [36]. For each article, the process involved familiarization with information, identification, recording, categorization and interpretation. We adapted Dolowitz and Marsh [15] policy transfer framework. The framework is based on six questions including: Why do actors engage in policy transfer? Who are the key actors involved in the policy transfer process? What is transferred? From where are lessons drawn? What are the different degrees of transfer? What restricts or facilitates the policy transfer process? and How is the process of policy transfer related to policy “success” or policy “failure”? [15] (p.8). In line with our study aim we focused on: Why engage in policy transfer? Who was engaged in the policy transfer? What was the role of actors and context in policy transfer? Did the policy transfer succeed or fail? - based on our definition indicated under study design and review questions sub-section.

## Results

### Overview

The article selection process is summarized in the flow chart (Fig. 1) while additional file 1 lists the searched databases, search dates and the yield. Out of 1114 citations, 9 articles satisfied the eligibility criteria after title, abstract, and full-length screening.

### Characteristics of included studies

The characteristics of the included studies in terms of country of study, year of publication, and health policy issue reported on are summarized in Table 1. Each of the nine articles included for analysis reports an empirical case of a health policy transfer from international to national level in a sub-Saharan Africa country. Two articles describe policy transfers in Malawi; one on hospital autonomy reforms [40] and the other one on health sector decentralization [39]. One article reports on policy transfer in Uganda, with another one studying both Uganda and Ghana [5, 41]. One article each reports a policy transfer case in Cameroon [38], Mozambique [37], South Africa [42] and Zambia [43]. One article describes policy transfer of integrated Community Case Management of Childhood illnesses (iCCM) in six [6] countries including Burkina Faso, Kenya, Malawi, Mali, Mozambique and Niger [30]. All policy issues described in the articles included in this study can be linked to a global or international policy agenda or issue (Table 1). These include health related MDGs [30, 37, 38, 42, 43], SDGs [5], World Bank Advanced structural adjustment programme (SAP) [39, 40] and World Health Assembly resolution on child dosage medicine formulations [41].

**Table 1:** Summary of key findings

***Policy/Country***	***Related global policy agenda***	***transfer mechanisms***	***Strategy***	***Successful or failed***
DOTS for TB and syndromic management for STIs in Mozambique [37]	MDGs	*Voluntary* through *Lessons drawing*	- Inter-country visits (visit to Zimbabwe to learn about syndromic management of STI) - Scientific and technical cross-national linkages between international players and the national stakeholders - Financial support	Successful
Global health policies for malaria and HIV/AIDS/ Cameroon [38]	MDGs	*Coercive: funding conditionality*	- Conditionality in accessing funding - Policy negotiation	Successful
Health sector decentralization in Malawi [39]	World Bank Structural Adjustment Program	*Coercive: funding conditionality*	- Conditionality on aid - Participatory learning and formal training - Bureaucratic bypass	Successful
Hospital autonomy reforms in Malawi [40]	World Bank Structural Adjustment Program	*Coercive*	- Conditionality on aid - Technical support	Failed
Child-appropriate dosage formulations policy from the global to national level/ Uganda [41]	WHO resolution (WHA60.20)	*Voluntary through lessons drawing*	- Policy negotiation - Technical support	Failed
Policy transfer in the context of the UNAIDS ‘90–90–90’ treatment targets in Ghana and Uganda [5]	SDGs	*Voluntary involving lessons drawing*	- Policy negotiation - Technical support - Financial support	Successful
Introduction of new molecular tuberculosis diagnostics in South Africa [42]	MDGs	*Voluntary involving lessons drawing*	- Technical support	Successful
Advocacy coalitions and the transfer of nutrition policy to Zambia [43]	MDGs	*Voluntary*	- Technical cross-national linkages between international players and the national stakeholders - Systematic advocacy - Technical assistance - Funding to the national drivers of the process	Successful
Understanding the role of international organizations in iCCM policy transfer in six African countries [30]	MDGs	*Mixed (voluntary and coercive) involving lessons drawing and conditional funding*	- Policy negotiation - Technical support (Provision of technical assistance) - Financial support (External funding targeted at iCCM) - International conferences - Publications in peer review journals - Study tours and global guidelines	Successful

From the review of the nine articles, the three major themes were policy transfer mechanisms, policy transfer strategy, and whether the transfer was successful or not. These are summarized in Table 1 and are elaborated below.

### Policy transfer mechanisms

Based on the reviewed literature, the main policy transfer mechanism in sub-Saharan Africa at least in the health sector is voluntary. This was discernable in five of the nine articles reviewed [5, 37, 41–43]. Three articles describe coercive policy transfers; the adoption of the global Roll Back Malaria (RBM) and the Accelerating Access Initiative (AAI) strategies into national policies and programs in Cameroon [38], hospital autonomy, and decentralization reforms in Malawi [39, 40]. One article describes a mixed policy transfer mechanism i.e., the adoption of iCCM policy in six countries [30].

### Policy transfer strategies

The policy transfer strategies especially where the transfer has been coercive was conditioning of development grants and loans on adoption of a global policy [38, 39] or initiating the process to adopt the global policy being advanced [40]. The adoption of the global Roll Back Malaria (RBM) and the Accelerating Access Initiative (AAI) strategies into national policies and programs in Cameroon were preconditions for accessing Global Fund and World Bank funding [38]. Similarly, Bender and colleagues [39] reporting on the health sector decentralization in Malawi also notes that “… because of international pressure and incentives, the Malawian politicians were very motivated to conduct the reform” (p.22). Also in Malawi. in order to realize hospital autonomy reform, United States Agency for International Development (USAID) conditioned its non-project assistance-based aid (NPA) on adoption of hospital autonomy.

Other prevalent policy transfer strategies were technical support or assistance by global stakeholders at national level [5, 30, 40–43], policy negotiations [5, 30, 38, 41], strong networking/linkages amongst national and global technical teams [37, 43], cross-country learning such as intercountry learning tours [30, 37, 39], keeping away other stakeholders from the process through ‘bureaucratic’ bypass [39] and dissemination of information to national level stakeholders on a policy issue either through supporting their participation in relevant global conferences or national dissemination workshops and publications [30, 39]. Even where the policy transfer mechanism was coercive through conditionality on development aid - policy negotiations, technical assistance, bureaucratic bypass etc. were part of the usually combined strategic approaches by the global stakeholders.

Therefore, the current study found that irrespective of the transfer mechanism, be it voluntary or coercion, there is need for a combination of policy transfer strategies as a single strategy may unlikely suffice. The policy transfer strategies can be applied to varying degrees depending on the issue and approach. These include: - peer learning through intercountry visits and conferences, cross-national linkages, financial support of the policy transfer process, conditionality on aid and technical assistance, competency building of national level stakeholders through participatory learning and formal training, systematic advocacy, bureaucratic maneuvering such as ‘bureaucratic bypassing’ and negotiations. However, better characterization of health policy issues and their likelihood of being successfully transferred or not from global to national level, as well as description and definition of policy transfer strategies are areas that need further scholarship and development.

### Policy transfer success or failure

Policy transfer in seven of the nine articles reviewed were successful as exemplified by adoption and/or development of national policies and strategies based on global agendas [5, 30, 37–39, 42, 43]. Two articles describe cases of failed policy transfer; the adoption of WHA resolution on child appropriate dosage formulations [41] and hospital autonomy reform in Malawi [40]. The success or failure of policy transfer seems to not necessarily be related to the mechanisms but a combination of the mix of strategies used, the actors involved and their inter-relationship and the contextual factors. For example, the Mozambique adoption its national policies and programs of the Directly Observed Therapy (DOTS) and syndromic management of Sexually Transmitted Infection (STI) were due to a mix of close networking between national actors; Ministry of Health (MOH) staff and international stakeholders including WHO, United Nations International Children’s Emergency Fund (UNICEF), Norwegian Agency for Development Cooperation (NORAD) and European Commission that provided the funding, and International Union Against TB and Lung Diseases (IUATLD) that provided technical support.

Similarly, successful policy transfer in Cameroon for Malaria and HIV/AIDS global strategies [38], health sectors decentralization reforms in Malawi [39], adoption of 90,90,90 AIDS target in Uganda and Ghana [5], new TB diagnostics in South Africa [42], nutrition policy in Zambia [43] and iCCM strategies in six Africa countries [30] were a combination of favorable contextual factors, actors level of influence and the policy transfer strategies mixes. The favorable contextual factors included epidemiological factors such high HIV and Malaria burden in Uganda, Ghana, South Africa and Cameroon; economic factors and low prioritization of health leading to reliance on external funding in most of the countries studied in the reviewed articles. Other factors were the strong coalition of international (United Nations Programme on HIV/AIDS (UNAIDS), World Bank, WHO, German Agency for Technical Cooperation (GTZ);now German Corporation for International Cooperation (GIZ), Department for International Development (DFID), USAID and European Commission, U.S. President’s Emergency Plan for AIDS Relief (PEPFAR)) and national stakeholders active in a given policy areas such as HIV and Malaria, [38, 42, 43], iCCM policy (the WHO, UNICEF, USAID, Save the Children and Ministries of Health) [30], nutrition policy (DFID, Irish Aid, Swedish International Development Cooperation Agency (SIDA), World Bank, European Union (EU) and USAID, Scaling Up Nutrition (SUN), MOH and National Central Statistics Organization) [43]; commitment on funding [30, 44]; policy negotiation [30]. Another facilitation factor for policy transfer is the the existence of institutional mechanisms anchored within MOH for dialogue such as the Central Technical Group and National Programmes Committees in Cameroon [38]. In addition, for certain policy issues such as adoption of the iCCM strategy, the iCCM policy, the readiness of health system was a key determinant [30]. For the case of iCCM, the ministries of health were also under pressure especially by politicians to deliver on the MDGs. The iCCM was seen as one of the key strategies to achieve child health related targets [30]. The nature of the policy issue within a given context is also a key determinant of the policy transfer process for example, nutrition in Zambia is not a politically sensitive issue [43], while hospital autonomy and decentralization are high political issues, hence the smooth process in Zambia compared to Malawi cases [39, 40].

The failed hospital autonomy reforms in Malawi was anticipated to lead to improved efficiency, effectiveness, quality and accountability [40]. Initially the political leadership agreed with the process of the reform [40] however as Tambulasi [40] notes, the initial commitment to adopt the hospital autonomy programme was only motivated by the desire to secure aid from the USAID NPA program. The proposed hospital autonomy reform was rejected at the Cabinet level, despite the initial commitment and large amount of resources spent on the policy transfer project [40]. These attests to the need for a mix of policy transfer strategies, understating of the country context and the motivation of actors.

Similarly, in Uganda as part of the better medicines for children program that followed the World Health Assembly resolution 60.20 (WHA60.20), the WHO member countries were to adopt and implement a policy on child appropriate dosage formulation. The policy transfer negotiations in Uganda did not result in the transfer of the WHA policy resolution on better medicines for children due to non-commitment by development partners on funding the initiative. The government stakeholders felt it would be a costly initiative and thus the child appropriate dosage formulations were not included in the national essential medicines list [41].

## Discussion

In this review, we explored how global health policies are transferred to national level in sub-Saharan African countries. Literature indicates that policy transfer in Lower and Middle Income Countries (LMIC) from global or international level to national level are predominantly coercive in nature [16]. However, our finding indicates that the common policy transfer process, at least in the health sector in sub-Saharan Africa are predominantly negotiated and/or voluntary. Six of the nine articles reviewed indicate that the transfers were voluntary or negotiated in nature involving policy dialogue and technical support. The health sector is complex and in sub-Saharan Africa, the health sector is largely funded by development partners. Therefore, one may argue that the health sector stewards are inherently programmed to accept international policies due to the perceived fear of losing funding as a sector, should they not support/or adopt international policy being advanced. However, the failed hospital autonomy reform in Malawi (country national budget is 50% externally funded [40]) attests otherwise. There is also the presumption that the international agents come with money as a coercing tool to national stakeholders. However, the case of iCCM policy adoption indicates that as part of the policy negotiation, the national stakeholders can condition acceptance of an international policy based on further funding support and in the process sending back the international agents to drawing boards on how to fund such policy initiatives. This approach of not fronting funding beforehand could also be due to the mounting criticism of international organizations of their approach on policy transfer to developing countries that has led to failures at policy implementation stage where the initial acceptance is based purely on the funding on offer [28, 45].

Marsh and Sharman [17] note that transfer mechanisms may operate concurrently and sometimes it may be difficult to distinguish which one is working during a particular policy transfer process. This is because it is possible for both voluntarism and coercion to operate concurrently as mechanisms during a particular global policy agenda transfer. This seems to have been the case in the iCCM policy transfer in the six countries [30] and the malaria and HIV policy transfers in Cameroon [38]. Therefore, in the current study, we confirm the notion of concurrent operation of mechanisms and in particular we make it clear that irrespective of the policy transfer mechanism, there is need for a right mix of policy transfer strategies. However, this area needs further exploration especially in terms of better characterizing or developing a framework for examining policy transfer strategies.

The current review confirms the critical role of actors and context in policy transfer as illustrated in both cases of policy transfer failure and success. Contextual factors such as epidemiologic factors are instrumental in policy transfer. They are important because in-country national policy makers would already be looking for possible policy solutions health conditions and they are likely to see policies being advanced by international organizations as best practices. This is discernable in the reviewed literature examining disease conditions; TB policies (Mozambique and South Africa) [37, 42], HIV/AIDS (Cameroon, Ghana and Uganda) [5, 38] and Malaria (Cameroon) [38], Nutrition (Zambia) [43]. For policies that are directed typically at specific health conditions, the epidemiological factors such as the high burden tend to favor successful transfer. This is because disease programs tend to be more technical areas with policy content from international level requiring majorly technical programming with limited political implications [43]. In sub-Saharan Africa, the disease programs of international interest tend to also be largely funded externally. However, where the required reforms are more systemic and require inputs beyond the health sector, such as enactment of Laws, the policy transfer process tends to be more difficult, whether voluntary or coercive. This can be seen in cases of decentralization and hospital autonomy reforms in Malawi (coercive processes) and the child dosage appropriate formulations in Uganda (voluntary process). Decentralization always has political implications and pharmaceutical supplies at national level involve multi-sectorial engagements than specific health condition policies and usually the stakes are higher given the amount of funding involved [46]. Therefore, stakeholders driving health financing reforms that advance UHC as recommended by the WHO need to better understand their political and bureaucratic environment given the wide-ranging systemic requirements of such reforms. In addition to epidemiological factors, other favorable factors for policy transfer in the health sector from global to national level in sub-Saharan Africa is the predominant reliance of external funding, existence of local platform or structure situated at and led or coordinated by MOH [47], good working relation between global and national level actors, high level political support and good understanding of the contextual factors by stakeholders driving the reforms.

## Conclusions

The divide between coercive and voluntary policy transfer mechanisms in sub-Saharan Africa requires more nuanced examination before one can conclusively say which mechanism is predominant. However, the current study indicates that even though the health sector is heavily donor depended in most sub-Saharan African countries, health policy transfer processes are generally negotiation-based and voluntary. National level stakeholders are receptive to international health policy agenda if it suits their interest and reject it if it does not, as the case of Malawi points out, and would reject reforms or make it impossible to implement if they can potentially lead to unfavorable political standing amongst the voters or make them loose their controlling power [39, 40, 43].

In sub-Saharan Africa, funding by development partners is key to the success of policy transfer, even where the transfer is voluntary. Nsabagasani et al. argues that even though it is the responsibility of member states to adopt WHA resolutions, the role of global influence, especially through funding of global health agendas are very important for the process of policy adoption and implementation at the national level [41].

Therefore, irrespective of the policy transfer mechanism that may operate for example in the transfer to national level of WHO health financing reform principles that advance UHC to achieve SDG 3, actors at all levels needs to take into account a number of considerations. Key amongst the factors that needs to be considered for a successful policy transfer include alignment with local need, understanding of context by global actors, existence of a national anchoring institution/platform for coordination and engagement, technical and financial support by the international actors, close linkages between international and national stakeholders, and limited potential effect of the policy on the bureaucratic structure and political status quo.

There is need to expand policy transfer studies to better define and characterized policy transfer strategies especially in sub-Saharan Africa where there is a lot of fluidity in the political and bureaucratic landscape. One key limitation of this study is that it did not explore how a policy will fare in terms of implementation once adopted from the Global level. The current study only defined success of policy transfer in terms of development of national policy or strategy document based on adoption of the global agenda and not in terms of implementation at the national level. Implementation of the policy once transferred to national from global level is an area that needs further exploration especailly amongst sub-Sharan African countries. The other limitation of this study is the few numbers of articles that were available for analysis. However, this is not surprising given that policy transfer studies especially in the health sector is an area with limited scholarship. Despite the limitations, this study makes contribution in terms of questioning the notion that transfers in Africa is predominantly voluntary and makes explicit the need for development of robust frameworks for examining policy transfer strategies.

## Supplementary Information

**Additional file 1.**

## Data Availability

The datasets used during the current study are available from the corresponding author on reasonable request.

## Abbreviations

AAI: Accelerating Access Initiative
CSO: Civil Society Organization
DCE: Development, Capable and Ethical State
DFID: Department for International Development
DOTS: Directly Observed Therapy
EU: European Union
GIZ: German Corporation for International Cooperation
GTZ: German Agency for Technical Cooperation
HIV: Human Immunodeficiency Virus
iCCM: Integrated Community Case Management of Childhood illnesses
LMIC: Lower and Middle Income Country
MDGs: Millennium Development Goals
NORAD: Norwegian Agency for Development Cooperation International
IUATLD: Union Against TB and Lung Diseases
NPA: Non-Project Assistance-Based Aid
PEPFAR: U.S. President’s Emergency Plan for AIDS Relief
RBM: Roll Back Malaria
SAP: Structural Adjustment Program
SDGs: Sustainable Development Goals
SHSPH: School of Health Systems and Public Health
SIDA: Swedish International Development Cooperation
STI: Sexually Transmitted Infection
SUN: Scaling Up Nutrition
TB: Tuberculosis
UHC: Universal Health Coverage
UNAIDS: United Nations Programme on HIV/AIDS
UNGA: United Nations General Assembly
UNICEF: United Nations International Children’s Emergency Fund
USAID: United States Agency for International Development
WHA: World Health Assembly
WHO: World Health Organization
