# Supplementary material for: How are global health policies transferred to sub-Saharan Africa countries? A systematic critical review of literature
Source: Global Health. 2022 Feb 23;18:25. doi: 10.1186/s12992-022-00821-9 (PMC8867733; doi:10.1186/s12992-022-00821-9)
Supplement: Supplementary file 1 — Additional file 1. [file 12992_2022_821_MOESM1_ESM.docx]

**Additional file 1**

**1. Tool for eligibility screening of articles**

***a) Screening for potential eligibility***

- Duplicate removal
- Title and abstract information screen against the following
  - Mention of policy transfer (or policy diffusion or lessons drawing) and health topic in the title that is/was a global issue

***b) Eligibility for inclusion for full-length articles screening for analysis***

- Describes in at least one of the article sections:
  - Empirical study on health policy transfer form global to national level
  - Policy transfer study on a global health agenda to a country in sub-Saharan Africa

**2. Results from the search strategy**

| **Database/ search engine** | **Date Searched** | **Hits** | **After Duplicate removal** | **Potentially eligible (Title/abstract screening)** | **Eligible**  **Full length screening** | **Eligible for analysis** |
| --- | --- | --- | --- | --- | --- | --- |
| Google scholar | 15 June 2021 | 10^ | 2114 | 978 | 44 | 9 |
| PubMed |  | 3160 |  |  |  |  |
| EBSCOhost* | 20 June 2021 | 529 |  |  |  |  |
| ProQuest | 25 July 2020 | 1412 |  |  |  |  |
| Scopus | 25 July 2020 | 249 |  |  |  |  |
| **Total** |  | **5350** | **2114** | **978** | **44** | **9** |

## *^ Potentially eligible (abstract) from google scholar*

## **EBSCO (CINAHL, EconLit, Health Source: Nursing/Academic Edition, Humanities Source, MEDLINE, APA PsycArticles, APA PsycInfo, Social Work Abstracts)*

**3. Example of Search string - PubMed**

*(((((policy transfer) OR (policy diffusion)) OR (policy convergence)) OR (policy learning)) AND (((((((sub-Saharan Africa) OR (Low income countr*)) OR (low and middle income countr*)) OR (LMIC)) OR (LIC)) OR (developing countr*)) OR ((((((((((((((((((((((((((((((((((((((((((((((((((Angola) OR (Benin)) OR (Botswana)) OR (Burkina Faso)) OR (Burundi)) OR (Cape Verde)) OR (Cameroon)) OR (Central African Republic)) OR (Chad)) OR (Comoros)) OR (Democratic Republic of the Congo)) OR (Republic of the Congo)) OR (Cote d'Ivoire)) OR (Djibouti)) OR (Equatorial Guinea)) OR (Eritrea)) OR (Ethiopia)) OR (Gabon)) OR (Gambia)) OR (Ghana)) OR (Guinea)) OR (Guinea-Bissau)) OR (Kenya)) OR (Lesotho)) OR (Liberia)) OR (Madagascar)) OR (Malawi)) OR (Mali)) OR (Mauritania)) OR (Mauritius)) OR (Mozambique)) OR (Namibia)) OR (Niger)) OR (Nigeria)) OR (Réunion)) OR (Rwanda)) OR (Sao Tome and Principe)) OR (Senegal)) OR (Seychelles)) OR (Sierra Leone)) OR (Somalia)) OR (South Africa)) OR (Sudan)) OR (Swaziland)) OR (Tanzania)) OR (Togo)) OR (Uganda)) OR (Western Sahara)) OR (Zambia)) OR (Zimbabwe)))) AND (health)*

**4. Data abstraction form**

**Reviewer: _______________________________ Date of data extraction:** ____________

**Bibliographic details of study (author. Year. Title.)**

_______________________________________________________________________

**Country of Study: _______________________________________________**

**Data Analysis**

- **Policy transfer mechanisms, process, strategies described _________________________________________________________**
- **Actors involved and their role in the policy transfer process _____________________________**
- **The contextual factors and their roles in the process**

**________________________________________________________________________**

**________________________________________________________________________**

**5. List of included study in the analysis**

1. McRobie E, Matovu F, Nanyiti A, Nonvignon J, Abankwah DNY, Case KK, et al. National responses to global health targets: Exploring policy transfer in the context of the unaids '90-90-90' treatment targets in ghana and uganda. Health Policy Plan. 2018; 33(1):17-33. doi:10.1093/heapol/czx132
2. Nsabagasani X, Hansen E, Mbonye A, Ssengooba F, Muyinda H, Mugisha J, et al. Explaining the slow transition of child-appropriate dosage formulations from the global to national level in the context of uganda: A qualitative study. Journal of Pharmaceutical Policy & Practice. 2015; 8(1):1-10. doi:10.1186/s40545-015-0039-1
3. Harris J. Advocacy coalitions and the transfer of nutrition policy to zambia. Health Policy Plan. 2019; 34(3):207-15. doi:10.1093/heapol/czz024
4. Tambulasi RIC. Conditionality, path dependence and veto points: The politics of hospital autonomy reforms in malawi. Journal of Asian & African Studies (Sage Publications, Ltd.). 2015; 50(2):176-95. doi:10.1177/0021909613515812
5. Bender K, Keller S, Willing H, editors. The role of international policy transfer and diffusion for policy change in social protection@ a review of the state of the art. 2015.
6. Colvin CJ, Leon N, Wills C, van Niekerk M, Bissell K, Naidoo P. Global-to-local policy transfer in the introduction of new molecular tuberculosis diagnostics in south africa. Int J Tuberc Lung Dis. 2015; 19(11):1326-38. doi:10.5588/ijtld.15.0262
7. Bennett S, Dalglish SL, Juma PA, Rodríguez DC. Altogether now... Understanding the role of international organizations in iccm policy transfer. Health policy and planning. 2015; 30 Suppl 2:ii26-ii35. doi:10.1093/heapol/czv071
8. Ngoasong MZ. Transcalar networks for policy transfer and implementation: The case of global health policies for malaria and hiv/aids in cameroon. Health Policy Plan. 2011; 26(1):63-72. doi:10.1093/heapol/czq018
9. Cliff J, Walt G, Nhatave I. What's in a name? Policy transfer in mozambique: Dots for tuberculosis and syndromic management for sexually transmitted infections. 2004. p. 38-55.
